# Untreated obstructive sleep apnea and accelerated cognitive decline over 10 years

**DOI:** 10.1002/alz.71533

**Published:** 2026-07-14

**Authors:** Christopher N. Kaufmann, Jennifer S. Albrecht, Vishaldeep Kaur Sekhon, Alden L. Gross, Emerson M. Wickwire, Halima Amjad, David L. Roth, Atul Malhotra, Marcela D. Blinka, Xiaowen Chen, Chien‐Yu Tseng, Marc Kaizi‐Lutu, Chunyu Liu, Kening Jiang, Ginger Chang, Adam P. Spira

**Affiliations:** ^1^ Department of Health Outcomes and Biomedical Informatics University of Florida College of Medicine Gainesville Florida USA; ^2^ Department of Epidemiology and Public Health University of Maryland School of Medicine Baltimore Maryland USA; ^3^ Division of Geriatric Medicine and Gerontology Johns Hopkins University School of Medicine Baltimore Maryland USA; ^4^ Center on Aging and Health Johns Hopkins University School of Medicine Baltimore Maryland USA; ^5^ Department of Epidemiology Johns Hopkins Bloomberg School of Public Health Baltimore Maryland USA; ^6^ Department of Psychiatry University of Maryland School of Medicine Baltimore Maryland USA; ^7^ Division of Pulmonary, Critical Care, and Sleep Medicine, Department of Medicine University of Maryland School of Medicine Baltimore Maryland USA; ^8^ Division of Pulmonary, Critical Care, and Sleep Medicine, Department of Medicine University of California San Diego School of Medicine La Jolla California USA; ^9^ Department of Mental Health Johns Hopkins Bloomberg School of Public Health Baltimore Maryland USA; ^10^ Department of Pharmaceutical Outcomes and Policy, College of Pharmacy University of Florida Gainesville Florida USA; ^11^ Department of Psychiatry and Behavioral Sciences Johns Hopkins School of Medicine Baltimore Maryland USA

**Keywords:** cognitive decline, continuous positive airway pressure, obstructive sleep apnea

## Abstract

**INTRODUCTION:**

Obstructive sleep apnea (OSA) is associated with cognitive decline, but short‐term studies show limited cognitive benefits of its treatment with continuous positive airway pressure (CPAP). We examined whether longer follow‐up exhibits greater cognitive differences associated with CPAP use.

**METHODS:**

We analyzed 777 participants from the 2011 National Health and Aging Trends Study (NHATS) with linked Medicare claims, with one or more claims for OSA and no baseline cognitive impairment. CPAP treatment was defined by one or more CPAP claims. Cognitive trajectories from 2011 to 2021 were estimated using a factor score derived from annual cognitive performance assessments and compared by CPAP treatment status using adjusted generalized linear mixed models.

**RESULTS:**

Cognitive performance declined over follow‐up. CPAP‐treated participants declined by −0.03 standard deviation (SD) units per year (95% confidence interval [CI]: −0.04, −0.02). Untreated participants experienced a 69% faster decline (CPAP‐by‐time interaction: −0.02; 95% CI: −0.04, −0.001).

**DISCUSSION:**

CPAP therapy may slow cognitive decline in older adults with OSA.

## Highlights

- Obstructive sleep apnea (OSA) is associated with cognitive decline.
- Studies show limited short‐term cognitive benefits of OSA treatment.
- However, long‐term effects of OSA treatment are unclear.
- We examined 10‐year cognitive trajectories in those with OSA by treatment status.
- Untreated OSA was associated with a faster cognitive decline than treated OSA.

## 1 BACKGROUND

Nearly one billion people worldwide have obstructive sleep apnea (OSA),^1^ a disorder characterized by repetitive pharyngeal collapse during sleep that increases the risk of cardiovascular disease and early death, while also contributing to higher healthcare use and reduced quality of life. In older adults, multiple epidemiologic studies have reported individuals with untreated OSA are more likely to develop dementia than those without OSA.^2^, ^3^, ^4^, ^5^, ^6^, ^7^ Several biologic pathways have been proposed to explain this link, including inflammation,^8^ oxidative stress,^9^, ^10^, ^11^ and sleep fragmentation.^12^ Moreover, past studies show the apolipoprotein E (*APOE)* ε4 allele is common in OSA, suggesting shared genetic susceptibility with Alzheimer's dementia.^13^, ^14^, ^15^ Given its high prevalence and strong potential biological mechanisms, untreated OSA stands out as a potentially important and impactful modifiable risk factor for dementia.

In this context, OSA is treatable and several efficacious therapies are available.^16^, ^17^, ^18^, ^19^, ^20^ The first line treatment recommended by the American Academy of Sleep Medicine, continuous positive airway pressure (CPAP), delivers continuous stream of air to maintain patency of the airway.^21^ Adherence to CPAP results in substantive improvements in OSA symptomatology,^16^, ^17^, ^18^, ^19^, ^20^ and importantly, is linked to downstream health improvements, including cardiometabolic and neuropsychiatric sequelae.^22^, ^23^, ^24^, ^25^, ^26^, ^27^ Although CPAP is efficacious, many individuals remain untreated,^28^, ^29^ which may prolong exposure to the pathophysiologic mechanisms (e.g., intermittent hypoxia and sleep fragmentation)^30^ driving the association between OSA and dementia. Failure to treat OSA may represent a missed opportunity to reduce dementia risk through a readily available intervention.

Observational studies suggest CPAP use may lower dementia risk.^31^, ^32^, ^33^, ^34^ In a cohort of US Medicare beneficiaries 65+ years with OSA, Dunietz et al. found treatment with CPAP was associated with decreased odds for incident Alzheimer's disease (AD) and dementia.^32^ Other studies have reported similar findings, supporting the association between OSA treatment and reduced dementia risk.^31^, ^32^, ^33^, ^34^ These studies use CPAP claims or usage as indicators of treatment exposure, indirectly suggesting that not using CPAP may be associated with higher dementia risk.

From a prevention standpoint, cognitive decline is a key marker of dementia risk. Despite evidence linking OSA treatment to lower dementia risk, findings on cognitive decline are mixed. Most studies show minimal short‐term cognitive differences between CPAP‐treated and untreated individuals.^35^, ^36^, ^37^, ^38^, ^39^, ^40^ The Apnea Positive Pressure Long‐term Efficacy Study (APPLES)—a multi‐center randomized controlled trial of > 1,000 patients with moderate to severe OSA—found only small improvements in cognitive performance from CPAP vs. sham‐CPAP after 6 months.^41^, ^42^, ^43^ While executive functioning improved slightly after 3 months, this did not sustain at month 6. However, cognitive decline is a gradual process unfolding over many years. Most studies have shorter follow‐up times and less frequent cognitive assessments, limiting their ability to capture subtle changes preceding dementia.^44^ Studies tracking cognitive changes over longer periods can detect earlier shifts before dementia and reveal if lack of treatment accelerates decline in later life, informing whether long‐term CPAP use offers protection. The lack of long‐term, repeated cognitive measurement in most existing studies represents a significant gap hindering our understanding of the cognitive health consequences of untreated OSA.

In the present study, we used data from the nationally representative National Health and Aging Trends Study and linked Medicare claims to examine the association of CPAP treatment with 10‐year cognitive trajectories among older adults with OSA. We hypothesized that, among individuals with OSA, those who did not receive CPAP treatment would experience a faster cognitive decline compared with those who received CPAP.

## 2 METHODS

### 2.1 Data source

We conducted a retrospective cohort study using data from the National Health and Aging Trends Study (NHATS; 2011 to 2021) and linked Center for Medicare and Medicaid Services (CMS) claims data from 2008 to 2021. These NHATS‐CMS linked data were accessed via the National Institute on Aging (NIA) LINKAGE program, a virtual secure enclave environment. NHATS is an ongoing study of a nationally representative sample of Medicare beneficiaries aged 65+ years initiated in 2011 and includes annual assessments of health and functioning. In 2015, a replenishment sample was recruited to account for participant attrition. NHATS survey data are publicly available (see http://www.nhats.org), and procedures to gain access to LINKAGE are available online (https://www.nia.nih.gov/research/dbsr/nia‐data‐linkage‐program‐linkage).

### 2.2 Study population

**FIGURE 1 alz71533-fig-0001:**
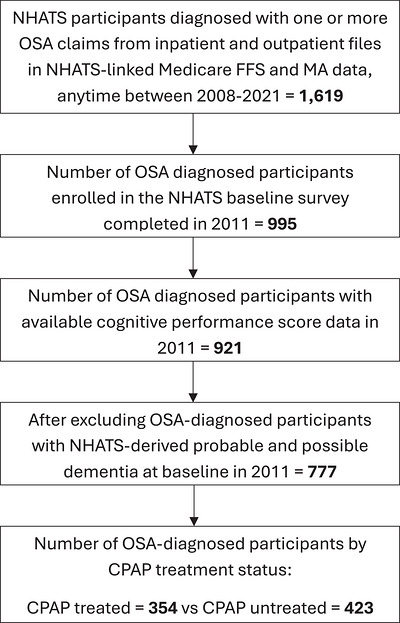
Flow diagram to identify NHATS participants with OSA. CPAP, continuous positive airway pressure; FFS, fee for service; MA, Medicare Advantage; NHATS, National Health and Aging Trends Study; OSA, obstructive sleep apnea.

Participants with one or more claims for OSA (see below) in Medicare data were considered for study inclusion. NHATS‐linked Medicare administrative data were extracted from inpatient, outpatient, durable medical equipment (DME), and master beneficiary summary files from the CMS Medicare fee‐for‐service (FFS) files from 2008‐2021, and Medicare Advantage (MA) files from 2015 to 2019. We limited our analysis to individuals who participated in the NHATS baseline survey (2011) and excluded participants who entered at the 2015 replenishment sample. Additionally, we excluded participants with prevalent possible or probable dementia in 2011 based on a validated algorithm,^45^ and those missing cognitive data at baseline. Of all linked claims data, we identified 1,619 with one or more OSA claims (*n* = 995 from 2011 baseline survey). After excluding individuals with prevalent possible or probable dementia and those missing cognitive data in 2011, our final analytical sample was *N* = 777. Please see Figure 1 for the flow diagram illustrating the creation of our cohort.

### 2.3 Measures

#### 2.3.1 OSA and CPAP

Participants with OSA were identified based on the presence of International Classification of Diseases (ICD), Version 9 codes 780.51, 327.23, 780.57, 780.53, or version 10 codes G47.33 and G47.30 in the inpatient and outpatient files of CMS FFS and MA data between 2008 and 2021. We required at least one diagnosis over the study period.

**RESEARCH IN CONTEXT**

1. **Systematic review**: The authors identified literature from PubMed, Google Scholar, and other related databases. The citations showed that obstructive sleep apnea (OSA) is associated with adverse cognitive outcomes, but there was mixed evidence of whether its treatment (predominantly with continuous positive airway pressure [CPAP]) protected against these outcomes. Specifically, although some large‐scale epidemiologic studies found a decreased risk of dementia diagnosis in CPAP users, smaller clinical trials showed only modest improvements in cognitive performance, with these studies typically lasting up to one year. These citations are cited in the manuscript.
2. **Interpretation**: The study shows that relative to those receiving CPAP, untreated OSA is associated with a faster decline in cognitive performance of up to 10 Model years. The findings suggest that extended follow‐up time is necessary to detect early cognitive decline linked to untreated OSA.
3. **Future directions**: The manuscript outlines several future directions for this research. These include mechanistic studies to identify pathways through which OSA treatment can prevent cognitive decline and long‐term observational studies connecting objective data from OSA treatment devices with medical claims. These studies would help clarify how CPAP adherence, intensity of use, and relative efficacy translate into physiological markers associated with cognitive decline.

CPAP treatment groups were identified by the Healthcare Common Procedure Coding System code (HCPCS) E0601 for CPAP device billing in the DME claims from 2008 to 2021. The presence of one or more claims with this HCPCS code identified CPAP‐treated participants; those without this code were considered CPAP‐untreated. We considered the CPAP treatment variable as time‐invariant, that is, if a claim was seen at any time between 2008 and 2021, the participant was categorized as CPAP‐treated during the entire study period.

#### 2.3.2 Cognitive performance

We[Fig alz71533-fig-0001] derived a longitudinally harmonized factor score for general cognitive performance based on cognitive tests administered in NHATS using item response theory methods.^46^ In NHATS interviews, participants were administered a brief set of cognitive tests in person. To evaluate memory, participants are administered a one‐trial‐immediate recall task of 10 nouns followed by a 5‐min delayed word recall. To evaluate orientation to time, participants were asked about the current month, day, year, and day of week. NHATS also asked participants to name the President and Vice President.^47^ This summary factor score for general cognitive performance has been shown to be more sensitive to changes than a simple average of immediate and delayed word recall from NHATS.^46^

#### 2.3.3 Covariates

We studied the covariates of age, sex, race/ethnicity, education, body mass index (BMI), marital status, physical activity, diabetes, stroke, heart disease, hypertension, anxiety, depression, insomnia symptoms, and smoking status from NHATS baseline (2011) data. Self‐reported physical activity was defined using two variables: whether in the past month, participants had ever gone for a walk for exercise, and whether they had performed any vigorous activities that increased their heart rate.^47^ Participants who reported no to both questions were considered not physically active, those who answered yes to walking but no to vigorous activity were considered having moderate physical activity, and those who answered yes to both were considered having vigorous physical activity. Marital status was not asked in the NHATS baseline year, so we used data from the following 4 years (2012–2015) to impute baseline marital status, which we categorized as married/partnered, separated/widowed/divorced, and never married. We calculated BMI using self‐reported height and weight from the baseline survey data, which we categorized as: underweight or normal (BMI < 25 kg/m^2^); overweight (BMI 25.0–29.9 kg/m^2^); and obese (BMI ≥30 kg/m^2^). Anxiety and depressive symptoms were evaluated by the Generalized Anxiety Disorder‐2 (GAD‐2) and Patient Health Questionnaire‐2 (PHQ‐2), and used a cut‐point of 3 or greater to indicate clinically significant symptoms.^48^ Smoking status was classified into past smokers, current smokers, and never smokers. Insomnia symptoms were determined based on two questions about whether, in the past month, participants took more than 30 min to fall asleep, and whether they woke up at night and had difficulty falling back to sleep, with a Likert scale ranging from every night to never. A response of “every night”, “most nights”, or “some nights” to either question was considered positive for insomnia symptoms. To address the missingness of BMI, education, marital status, stroke, hypertension, and heart disease in the data, multiple imputations using chained equations (*n* = 10) were performed.

### 2.4 Statistical analysis

We first summarized baseline characteristics using means and standard deviation for continuous variables and frequencies and percentages for categorical variables. We tested differences in baseline characteristics between groups using Student's *t*‐tests and chi‐squared tests.

To estimate the association of CPAP treatment with change in cognitive performance over time, we used linear mixed effects models.^49^ We built an unadjusted model with fixed effects for CPAP treatment group, time (years since 2011), and the interaction of CPAP and time, and random effects for participant and time. Next, we adjusted for covariates associated with CPAP treatment at *p* ≤0.2 in bivariate analysis that were not considered to be in the causal pathway between OSA and cognitive decline (i.e., age, BMI, education, and marital status). A third model added all covariates assessed, most of which are potentially in the causal pathway (i.e., age, sex, race, BMI, education, physical activity, marital status, stroke, heart disease, hypertension, diabetes, depression, anxiety, insomnia symptoms, and smoking status). Because our definition of OSA treatment was one or more claims for CPAP, which might not indicate continuous use, we also performed a sensitivity analysis. We repeated the models but compared trajectories for those with no CPAP charges (PAP non‐initiation), one to three charges (PAP partial adherence), and four or more charges (PAP adherence) to see if the results were affected by this categorization. This approach is consistent with Medicare policy for PAP adherence and prior research studies using Medicare claims.^50^, ^51^

To visualize cognitive trajectories, we estimated the marginal outcome (cognitive performance) from adjusted linear mixed models and plotted these values against years since 2011 by CPAP‐treatment group. Our study was approved by the University of Florida Institutional Review Board with reliance agreements at Johns Hopkins University and the University of Maryland, Baltimore. Although NHATS offers survey design and weighting variables for nationally representative estimates, we opted not to include them since our focus was solely on the specific group of older adults with OSA, not on creating nationally representative estimates. All analyses were performed using SAS 9.4 edition.

## 3 RESULTS

Table 1 displays demographic and clinical characteristics of participants with diagnosed OSA. Of these 777 participants, 354 (46%) had at least one claim for CPAP (CPAP‐treated group), and 423 (54%) received no claim for CPAP over 11 years (2011–2021). CPAP‐treated participants were slightly younger than CPAP‐untreated participants (mean [standard deviation {SD}] = 74.17 [5.68] vs. 75.32 [6.75] years, *p* = 0.01). They were also more educated, with 59% of them having some college or more compared to 51% of the CPAP‐untreated group (*p *= 0.03). CPAP‐treated participants were more likely to be married or partnered compared to CPAP‐untreated participants (66% vs. 52%, *p *= 0.001). Compared to CPAP‐treated participants, insomnia symptoms and anxiety were significantly higher among CPAP‐untreated participants (insomnia symptoms: 33% vs. 26%, *p *= 0.02; anxiety: 15% vs. 9%, *p *= 0.01). At baseline, CPAP‐treated participants had on average a higher harmonized factor score for cognitive tests compared to CPAP‐untreated participants (−0.17 [0.63] vs. −0.32 [0.63], *p *= 0.001).

**TABLE 1:** Baseline characteristics of NHATS participants diagnosed with OSA by CPAP use.

	Overall (*N* = 777)	CPAP Treated (*n* = 354)	CPAP Untreated (*n* = 423)	*p*‐value^1^
Age, mean (SD)	74.80 (6.31)	74.17 (5.68)	75.32 (6.75)	0.01
Baseline cognition factor score, mean (SD)	−0.25 (0.63)	−0.17 (0.63)	−0.32 (0.63)	0.001
**Sex, *n* (%)**				0.90
Female	391 (50.32)	179 (50.56)	212 (50.12)	
Male	386 (49.68)	175 (49.44)	211 (49.88)	
**Race/ethnicity, *n* (%)**				0.38
Non‐Hispanic Black	162 (20.85)	66 (18.64)	96 (22.70)	
Non‐Hispanic White	560 (72.07)	263 (74.29)	297 (70.21)	
Hispanic and Others	55 (7.08)	25 (7.06)	30 (7.09)	
**Education, *n* (%)**				0.03
Less than high school	141 (18.15)	50 (14.12)	92 (21.75)	
High school diploma or equivalent	212 (27.28)	96 (27.12)	115 (21.79)	
Some college	221 (28.44)	103 (29.10)	118 (27.90)	
College degree or higher	203 (26.13)	105 (29.66)	98 (23.17)	
**BMI, *n* (%)**				0.07
Normal/underweight	94 (12.1)	34 (9.60)	60 (14.18)	
Overweight	266 (34.23)	117 (33.05)	149 (35.22)	
Obese	417 (53.67)	203 (57.34)	214 (50.59)	
**Marital status, *n* (%)**				< 0.001
Married/partner	453 (58.3)	232 (65.54)	220 (52.01)	
Separated/widow/never married	324 (41.7)	122 (34.46)	203 (47.99)	
**Physical activity, *n* (%)**				0.43
No to limited	248 (31.92)	106 (29.94)	142 (33.57)	
Moderate	222 (28.57)	100 (28.25)	122 (28.84)	
Vigorous	307 (39.51)	148 (41.81)	159 (37.59)	
**Diabetes, *n* (%)**				0.94
Yes	271 (34.88)	123 (34.75)	148 (34.99)	
No	506 (65.12)	231 (65.25)	275 (65.01)	
**Stroke, *n* (%)**				0.44
Yes	100 (12.87)	42 (11.86)	58 (13.71)	
No	677 (87.13)	312 (88.14)	365 (86.29)	
**Heart disease, *n* (%)**				0.27
Yes	199 (25.61)	84 (23.73)	115 (27.19)	
No	578 (74.39)	270 (76.27)	308 (72.81)	
**Hypertension, *n* (%)**				0.86
Yes	586 (75.42)	268 (75.71)	318 (75.18)	
No	191 (24.58)	86 (24.29)	105 (24.82)	
**Anxiety, *n* (%)**				
Yes	98 (12.61)	33 (9.32)	65 (15.37)	0.01
No	679 (87.39)	321 (90.68)	358 (84.63)	
**Depression, *n* (%)**				
Yes	103 (13.26)	39 (11.02)	64 (15.13)	0.09
No	674 (86.74)	315 (88.98)	359 (84.87)	
**Insomnia symptoms, *n* (%)**				
Yes	232 (29.86)	91 (25.71)	141 (33.33)	0.02
No	545 (70.14)	263 (74.29)	282 (66.67)	
**Smoking, *n* (%)**				
Current smoker	60 (7.72)	20 (5.65)	40 (9.46)	0.13
Past smoker	390 (50.19)	179 (50.56)	211 (49.88)	
Never smoker	327 (42.08)	155 (43.79)	172 (40.66)	

^1^ *t*‐test for continuous variables, chi‐square test for categorical variables.

Abbreviations: BMI, body mass index; CPAP, continuous positive airway pressure; NHATS, National Health and Aging Trends Study; OSA, obstructive sleep apnea; SD, standard deviation.

In the unadjusted model (Model 1), the rate of change in cognitive test performance among CPAP‐treated participants was −0.03 SD units per year, while the rate of change among CPAP‐untreated participants was steeper at −0.05 SD units per year (Table 2). The difference in rate of cognitive decline comparing CPAP‐untreated to CPAP‐treated participants was statistically significant (Model 1: β = −0.02, 95% confidence interval [CI] = −0.04, −0.001), indicating that, compared to participants in the CPAP‐treated group, those in the CPAP‐untreated group had a 69% faster subsequent decline in cognitive performance. The significance of the difference in rate of cognitive decline was robust to adjustment for age, education, BMI, and marital status (Model 2: β = −0.02, 95% CI = −0.04, −0.001) as well as to hypothesized mediators (Model 3: β = −0.02, 95% CI = −0.04, −0.002). Please see supplementary material for model variance estimates (Tables S1 and S2).

**TABLE 2:** Association between CPAP treatment and cognitive performance score among NHATS participants diagnosed with OSA.

	Model 1		Model 2		Model 3	
	**Coefficient (95% CI)**	***p*‐value**	**Coefficient (95% CI)**	***p*‐value**	**Coefficient (95% CI)**	***p*‐value**
CPAP: Untreated vs treated (ref)	−0.125 (−0.209, −0.042)	0.003	−0.042 (−0.116, 0.033)	0.271	−0.018 (−0.009, 0.054)	0.627
Time (per year)	−0.027 (−0.040, −0.015)	<0.001	−0.027 (−0.039, −0.015)	<0.001	−0.027 (−0.039, −0.015)	<0.001
Interaction: CPAP × time	−0.019 (−0.037, −0.001)	0.04	−0.019 (−0.036, −0.001)	0.034	−0.019 (−0.036, −0.002)	0.032

*Note*: Model 1: Unadjusted linear mixed model for cognitive performance score with CPAP × time interaction. Model 2: Adjusted for age, BMI, education, and marital status with CPAP × time interaction. Model 3: Adjusted for age, sex, race, BMI, education, physical activity, marital status, stroke, heart disease, hypertension, diabetes, depression, anxiety, insomnia symptoms, and smoking status, with CPAP x time interaction.

Abbreviations: BMI, body mass index; CI, confidence interval; CPAP, continuous positive airway pressure; NHATS, National Health and Aging Trends Study; OSA, obstructive sleep apnea.

**FIGURE 2:**
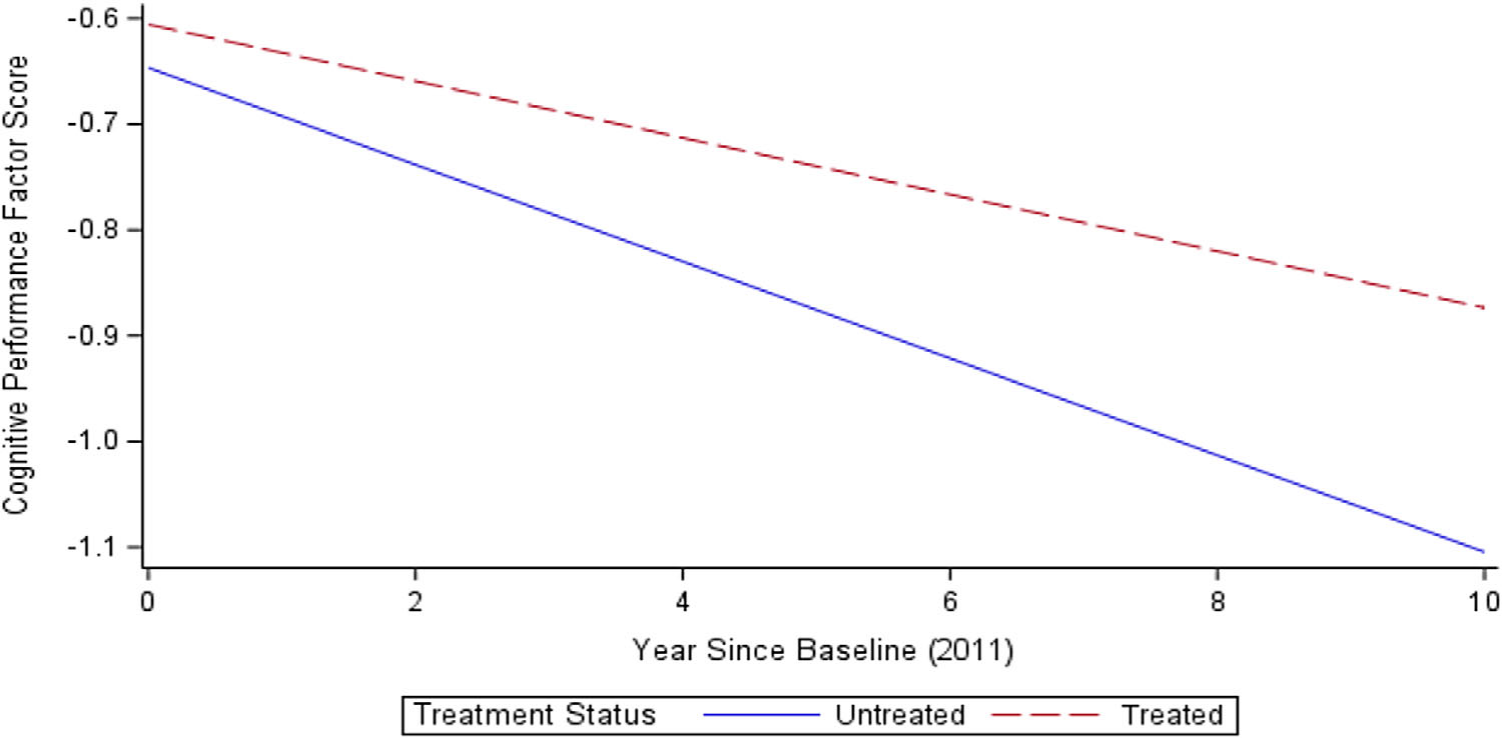
Marginal effects of CPAP treatment on cognitive performance trajectories between 2011 and 2021 among NHATS participants with OSA diagnosis (Model 2; *n* = 777). The X‐axis corresponds to the number of years since study entry at the 2011 NHATS interview. Fit computed at age = 74.2 years; education, less than high school; BMI, underweight/normal; and marital status, never married/separated/widowed. BMI, body mass index; CPAP, continuous positive airway pressure; NHATS, National Health and Aging Trends Study; OSA, obstructive sleep apnea.

Figure 2 displays the estimated trajectories of cognitive decline for CPAP‐treated and CPAP‐untreated participants from regression Model 2. On average, CPAP‐untreated participants had lower cognitive scores at baseline and experienced faster cognitive decline over time compared to CPAP‐treated participants.

In sensitivity analyses, of the *n* = 354 CPAP‐treated participants, the majority (85%; *n* = 300) had four or more CPAP charges. In all models, relative to those with four or more CPAP charges, there was a significantly faster decline in those with no CPAP charges (Model 2: β = −0.02, 95% CI = −0.04, −0.0008; Model 3: β = −0.02, 95% CI = −0.04, −0.0009). The decline observed in the one to four CPAP charges group was not statistically significant but was in the negative direction (data not shown).

## 4 DISCUSSION

In this nationally representative observational cohort, we found that individuals with OSA who did not receive CPAP had a 69% faster decline in cognitive performance compared with those who received CPAP over up to 10 years. The findings were robust, even after adjustment for cardiovascular disease and insomnia, which are themselves risk factors for dementia and are associated with OSA.^52^, ^53^ Taken together, our results suggest that treating OSA with CPAP may be critical for maintaining cognitive health in later life.

Our findings align with several large observational studies that compared individuals with and without CPAP treatment, showing lower dementia risk among those treated.^31^, ^32^, ^33^, ^34^ The present study adds to this body of research in several ways. First, our study included annual cognitive assessments over a 10‐year period, enabling the construction of cognitive trajectories with high temporal resolution over a long period. This contrasts with smaller clinical trials, most of which have a maximum follow‐up of only 1 year.^35^, ^36^, ^37^, ^38^, ^39^, ^40^, ^41^, ^42^, ^43^ While clinical trials have not consistently demonstrated short‐term cognitive benefits of CPAP, our long‐term observational results suggest that lack of CPAP treatment is associated with accelerated cognitive decline, which may only become detectable over extended follow‐up. Additionally, our study integrated Medicare claims data, self‐reported health behavior data, and objective cognitive performance data, providing unique insights. In contrast, earlier studies that only used Medicare claims data did not assess cognitive performance, thus failing to capture all but the most severe cognitive decline. Of note, by linking self‐reported data with Medicare claims, we were able to control for important covariates typically unavailable in claims‐based analyses. For example, we adjusted for physical activity and BMI, which are significant confounders for the OSA‐cognitive decline association, but are not captured or are inadequately documented in claims data alone. This approach strengthens the validity of our findings.

A number of potential mechanisms for our observed findings should be considered.^7^ First, OSA is characterized by chronic intermittent hypoxia with re‐oxygenation leading to oxidative stress.^54^ Oxidative stress has been clearly implicated in Alzheimer's disease and related dementias (ADRD) pathogenesis. Intermittent hypoxia can also trigger apoptosis in the hippocampus and thus some of the hypoxia‐induced effects may not be readily reversible^55^ for example, once brain atrophy is established. CPAP consistently leads to resolution of hypoxemia and, thus, would be predicted to mitigate the neurological impact of intermittent hypoxia.^56^ Second, amyloid and tau are core features of AD.^57^ Notably, glymphatic expansion during sleep has been hypothesized to facilitate clearance of toxins, including amyloid, which may be disrupted in the context of sleep fragmentation.^58^ Thus, the improvement in sleep quality more broadly with CPAP therapy may reduce markers of ADRD risk. Third, a randomized trial showed that CPAP treatment can lead to improvement in sleep‐dependent memory consolidation compared to usual care in people with OSA.^59^, ^60^ These findings suggest improvement in memory and may thus be another proposed mechanism explaining our findings. Further research is warranted to evaluate these mechanisms over longer follow‐up periods.

Our study has important clinical implications. The detection, diagnosis, and treatment of OSA may be an important strategy to preserve cognitive health and potentially prevent dementia. Because untreated OSA was associated with substantially faster cognitive decline, improving detection and ensuring that individuals initiate and maintain CPAP treatment may help preserve cognitive function as people age. Indeed, with the aging of the population and projected increases in the number of individuals affected by dementia,^61^ the need for effective interventions becomes even more critical. Our results suggest that addressing modifiable risk factors, such as a highly prevalent condition like OSA,^1^ should be a central part of strategies to slow cognitive decline. However, it is important to note that many patients struggle with long‐term adherence to CPAP therapy.^62^, ^63^ While Medicare requires clinicians to submit CMS proof showing that patients actually use CPAP for reimbursement,^64^ adherence continues to be a challenge that is not sufficiently documented in claims.^65^ This gap should be a focus for future research and clinical practice, especially when considering cognitive outcomes. Ultimately, improving both diagnosis and sustained engagement with OSA treatment could have substantial benefits for cognitive outcomes in the aging population.

The therapeutic landscape for OSA is rapidly evolving.^66^ In addition to CPAP alternatives, including mandibular advancement devices and surgical interventions,^29^ glucagon‑like peptide‑1 receptor agonists and related weight loss medications are emerging as new treatment options for the condition.^67^, ^68^ These developments call for more studies comparing the effectiveness of various OSA therapeutic options and how treatment decisions affect related outcomes, including cognition.

This study has several notable strengths, including the use of a large, population‐based cohort of older adults with 10 years of annual cognitive assessments linked to Medicare claims, which allowed for robust evaluation of cognitive trajectories, identification of OSA and treatment status, and access to self‐reported lifestyle data. However, several limitations should be acknowledged. Most importantly, our observational design cannot establish causality. Some participants may have initiated CPAP well into their cognitive trajectory, and other factors, such as OSA severity, adherence, or comorbidities, could also play a role. Thus, we support the design of future mechanistic clinical trials which could be informed by our new findings. Second, our identification of OSA was based on administrative Medicare claims, which we were unable to confirm with sleep laboratory results. A related point is that we were unable to assess OSA symptoms such as sleepiness, which have been associated with adverse OSA outcomes. Although it should be noted that results were similar in Model 3, which included adjustment for “insomnia symptoms.” Third, we were unable to directly assess adherence to CPAP therapy and instead relied on a claim to indicate treatment. This approach cannot capture sustained use or patterns of adherence. We treated CPAP as a time‐fixed variable to examine whether any evidence of use was associated with cognitive trajectories, but this does not reflect overall or continued use and may have influenced the results. We treated CPAP exposure as time‑invariant because preliminary analyses indicated that approximately half of the participants had evidence of CPAP use prior to the start of the study (2011 NHATS interview). As a result, individuals using CPAP before study entry would represent a heterogeneous group with varying cumulative exposure to CPAP. Additionally, due to CMS reimbursement rules, sustained use beyond 13 months of CPAP machine reimbursement is challenging to determine. It should be noted that the majority of patients had four or more claims, and there was still a statistically significant decline in cognitive performance in those with no CPAP charges compared to these individuals. Fourth, other therapies for OSA, such as mandibular advancement devices or surgery, are not captured in Medicare claims, and it is possible that participants in the untreated‐CPAP group were using these therapies. However, CPAP is the most common treatment for OSA and is considered the first‐line therapy, so we believe this limitation and any potential misclassification is unlikely to have substantially affected our findings. Fourth, our cognitive assessments focused solely on global cognition, as the available measures in NHATS did not permit evaluation of specific cognitive domains. Different cognitive domains, such as memory and executive functioning, may follow distinct trajectories as a function of OSA. Future research is needed to examine these differences in greater detail. Fifth, although we adjusted for many potential confounders, unmeasured confounding remains possible. However, our analytic approach sought to address residual confounding as comprehensively as possible through adjustment of these variables.

In summary, our findings demonstrate that untreated OSA is associated with a 69% faster rate of cognitive decline over a decade, reinforcing the importance of early intervention and recognizing OSA as a key risk factor for dementia. By managing OSA, clinicians have the opportunity to meaningfully impact long‐term cognitive health and contribute to dementia prevention as the population ages. Moving forward, continued research in this area will be essential to fully understand the scope of OSA treatment's cognitive benefits and to inform future clinical and public health strategies for protecting brain health in older adults.

## CONFLICT OF INTEREST STATEMENT

Christopher N. Kaufmann has previously served as a paid consultant to Jazz Pharmaceuticals. Atul Malhotra is funded by NIH. He reports income from Livanova, Eli Lilly, Powell Mansfield, Zoll and Sunrise. He is co‐founder of Clairyon unrelated to this topic. Resmed gave a philanthropic donation to UCSD. Emerson M. Wickwire's institution has received research funding from the AASM Foundation, Department of Defense, Merck, NIH/NIA, ResMed, the ResMed Foundation, and the SRS Foundation. Emerson M. Wickwire has served as a scientific consultant to Axsome Therapeutics, DayZz, Eisai, EnsoData, Idorsia, Merck, Nox Health, Primasun, Purdue, and ResMed and is an equity shareholder in WellTap. Adam P. Spira is supported in part by grants from the National Institutes of Health/National Institute on Aging. He has served as a consultant to Sequoia Neurovitality, BellSant, Inc., Amissa, Inc., and Synaptic Health, LLC. All other authors report no conflict of interest. Author disclosures are available in the Supporting Information.

## CONSENT STATEMENT

All human subjects in the National Health and Aging trends study provided informed consent.

## Supporting information




Supporting Information

Supporting Information

## References

[1] Benjafield AV , Ayas NT , Eastwood PR , et al. Estimation of the global prevalence and burden of obstructive sleep apnoea: a literature‐based analysis. Lancet Respir Med. 2019;7(8):687‐698. doi:10.1016/S2213-2600(19)30198-5 31300334 PMC7007763

[2] Ancoli‐Israel S . Epidemiology of sleep disorders. Clin Geriatr Med. 1989;5(2):347‐362. 2665916

[3] Ancoli‐Israel S , Klauber MR , Butters N , Parker L , Kripke DF . Dementia in institutionalized elderly: relation to sleep apnea. J Am Geriatr Soc. 1991;39(3):258‐263. doi:10.1111/j.1532-5415.1991.tb01647.x 2005339

[4] Ancoli‐Israel S , Klauber MR , Kripke DF , Parker L , Cobarrubias M . Sleep apnea in female patients in a nursing home. Increased risk of mortality. Chest. 1989;96(5):1054‐1058. doi:10.1378/chest.96.5.1054 2805836

[5] Bliwise DL , Yesavage JA , Tinklenberg JR , Dement WC . Sleep apnea in Alzheimer's disease. Neurobiol Aging. 1989;10(4):343‐346. doi:10.1016/0197-4580(89)90046-8 2812195

[6] Sharma RA , Varga AW , Bubu OM , et al. Obstructive sleep apnea severity affects amyloid burden in cognitively normal elderly. A longitudinal study. Am J Respir Crit Care Med. 2018;197(7):933‐943. doi:10.1164/rccm.201704-0704OC 29125327 PMC6020410

[7] Yaffe K , Laffan AM , Harrison SL , et al. Sleep‐disordered breathing, hypoxia, and risk of mild cognitive impairment and dementia in older women. JAMA. 2011;306(6):613‐619. doi:10.1001/jama.2011.1115 21828324 PMC3600944

[8] Ercolano E , Bencivenga L , Palaia ME , et al. Intricate relationship between obstructive sleep apnea and dementia in older adults. GeroScience. 2024;46(1):99‐111. doi:10.1007/s11357-023-00958-4 37814196 PMC10828345

[9] Lavie L . Obstructive sleep apnoea syndrome—an oxidative stress disorder. Sleep Med Rev. 2003;7(1):35‐51. doi:10.1053/smrv.2002.0261 12586529

[10] Suzuki YJ , Jain V , Park AM , Day RM . Oxidative stress and oxidant signaling in obstructive sleep apnea and associated cardiovascular diseases. Free Radic Biol Med. 2006;40(10):1683‐1692. doi:10.1016/j.freeradbiomed.2006.01.008 16678006 PMC1995030

[11] Yamauchi M , Nakano H , Maekawa J , et al. Oxidative stress in obstructive sleep apnea. Chest. 2005;127(5):1674‐1679. doi:10.1378/chest.127.5.1674 15888845

[12] Kang JE , Lim MM , Bateman RJ , et al. Amyloid‐beta dynamics are regulated by orexin and the sleep‐wake cycle. Science. 2009;326(5955):1005‐1007. doi:10.1126/science.1180962 19779148 PMC2789838

[13] Gottlieb DJ , Destefano AL , Foley DJ , et al. APOE ε4 is associated with obstructive sleep apnea/hypopnea. Neurology. 2004;63:664‐668. 15326239 10.1212/01.wnl.0000134671.99649.32

[14] Osorio RS , Ayappa I , Mantua J , et al. Interaction between sleep‐disordered breathing and apolipoprotein E genotype on cerebrospinal fluid biomarkers for Alzheimer's disease in cognitively normal elderly individuals. Neurobiol Aging. 2014;35(6):1318‐1324. doi:10.1016/j.neurobiolaging.2013.12.030 24439479 PMC4022140

[15] Uyrum E , Balbay O , Annakkaya AN , Gulec Balbay E , Silan F , Arbak P . The relationship between obstructive sleep apnea syndrome and apolipoprotein E genetic variants. Respiration. 2015;89(3):195‐200. doi:10.1159/000369560 25613112

[16] Montesi SB , Edwards BA , Malhotra A , Bakker JP . The effect of continuous positive airway pressure treatment on blood pressure: a systematic review and meta‐analysis of randomized controlled trials. J Clin Sleep Med. 2012;8(5):587‐596. doi:10.5664/jcsm.2170 23066375 PMC3459209

[17] Patel SR , White DP , Malhotra A , Stanchina ML , Ayas NT . Continuous positive airway pressure therapy for treating sleepiness in a diverse population with obstructive sleep apnea: results of a meta‐analysis. Arch Intern Med. 2003;163(5):565‐571. doi:10.1001/archinte.163.5.565 12622603

[18] Pepperell JC , Ramdassingh‐Dow S , Crosthwaite N , et al. Ambulatory blood pressure after therapeutic and subtherapeutic nasal continuous positive airway pressure for obstructive sleep apnoea: a randomised parallel trial. Lancet. 2002;359(9302):204‐210. doi:10.1016/S0140-6736(02)07445-7 11812555

[19] Schwartz M , Acosta L , Hung YL , Padilla M , Enciso R . Effects of CPAP and mandibular advancement device treatment in obstructive sleep apnea patients: a systematic review and meta‐analysis. Sleep Breath. 2018;22(3):555‐568. doi:10.1007/s11325-017-1590-6 29129030

[20] Weaver TE , Mancini C , Maislin G , et al. Continuous positive airway pressure treatment of sleepy patients with milder obstructive sleep apnea: results of the CPAP Apnea Trial North American Program (CATNAP) randomized clinical trial. Am J Respir Crit Care Med. 2012;186(7):677‐683. doi:10.1164/rccm.201202-0200OC 22837377 PMC3480519

[21] Battan G , Kumar S , Panwar A , et al. Effect of CPAP therapy in improving daytime sleepiness in Indian patients with moderate and severe OSA. J Clin Diagn Res. 2016;10(11):OC14‐OC16. doi:10.7860/JCDR/2016/23800.8876 PMC5198373 28050420

[22] Xu PH , Fong DYT , Lui MMS , Lam DCL , Ip MSM . Cardiovascular outcomes in obstructive sleep apnoea and implications of clinical phenotyping on effect of CPAP treatment. Thorax. 2023;78(1):76‐84. doi:10.1136/thoraxjnl-2021-217714 35304425 PMC9763161

[23] Sateia MJ . Neuropsychological impairment and quality of life in obstructive sleep apnea. Clin Chest Med. 2003;24(2):249‐259. doi:10.1016/s0272-5231(03)00014-5 12800782

[24] Wickwire EM , Bailey MD , Somers VK , et al. CPAP adherence is associated with reduced inpatient utilization among older adult Medicare beneficiaries with pre‐existing cardiovascular disease. J Clin Sleep Med. 2021;18:39‐45. doi:10.5664/jcsm.9478 PMC8807906 34170251

[25] Wickwire EM , Bailey MD , Somers VK , et al. CPAP adherence reduces cardiovascular risk among older adults with obstructive sleep apnea. Sleep Breath. 2021;25(3):1343‐1350. doi:10.1007/s11325-020-02239-2 33141315

[26] Wickwire EM , Bailey MD , Somers VK , et al. CPAP adherence is associated with reduced risk for stroke among older adult Medicare beneficiaries with obstructive sleep apnea. J Clin Sleep Med. 2021;17(6):1249‐1255. doi:10.5664/jcsm.9176 33612161 PMC8314664

[27] Wickwire EM , Lettieri CJ , Cairns AA , Collop NA . Maximizing positive airway pressure adherence in adults: a common‐sense approach. Chest. 2013;144(2):680‐693. doi:10.1378/chest.12-2681 23918114

[28] Djonlagic I , Guo M , Matteis P , Carusona A , Stickgold R , Malhotra A . Untreated sleep‐disordered breathing: links to aging‐related decline in sleep‐dependent memory consolidation. PLoS One. 2014;9(1):e85918. doi:10.1371/journal.pone.0085918 24489679 PMC3906012

[29] Faria A , Allen AH , Fox N , Ayas N , Laher I . The public health burden of obstructive sleep apnea. Sleep Sci. 2021;14(3):257‐265. doi:10.5935/1984-0063.20200111 35186204 PMC8848533

[30] Lal C , Ayappa I , Ayas N , et al. The link between obstructive sleep apnea and neurocognitive impairment: an official American thoracic society workshop report. Ann Am Thorac Soc. 2022;19(8):1245‐1256. doi:10.1513/AnnalsATS.202205-380ST 35913462 PMC9353960

[31] Cho JH , Suh JD , Han KD , Jung JH , Lee HM . Uvulopalatopharyngoplasty may reduce the incidence of dementia caused by obstructive sleep apnea: national insurance service survey 2007‐2014. J Clin Sleep Med. 2018;14(10):1749‐1755. doi:10.5664/jcsm.7388 30353808 PMC6175811

[32] Dunietz GL , Chervin RD , Burke JF , Conceicao AS , Braley TJ . Obstructive sleep apnea treatment and dementia risk in older adults. Sleep. 2021;44(9):zsab076. doi:10.1093/sleep/zsab076 33769542 PMC8436135

[33] Osorio RS , Gumb T , Pirraglia E , et al. Sleep‐disordered breathing advances cognitive decline in the elderly. Neurology. 2015;84(19):1964‐1971. doi:10.1212/WNL.0000000000001566 25878183 PMC4433459

[34] Tsai MS , Li HY , Huang CG , et al. Risk of Alzheimer's disease in obstructive sleep apnea patients with or without treatment: real‐world evidence. Laryngoscope. 2020;130(9):2292‐2298. doi:10.1002/lary.28558 32045010

[35] Costa YS , Lim ASP , Thorpe KE , et al. Investigating changes in cognition associated with the use of CPAP in cognitive impairment and dementia: a retrospective study. Sleep Med. 2023;101:437‐444. doi:10.1016/j.sleep.2022.11.037 36516600

[36] Crawford‐Achour E , Dauphinot V , Martin MS , et al. Protective effect of long‐term CPAP therapy on cognitive performance in elderly patients with severe OSA: the PROOF study. J Clin Sleep Med. 2015;11(5):519‐524. doi:10.5664/jcsm.4694 25700873 PMC4410925

[37] Kouri I , Kolla BP , Morgenthaler TI , Mansukhani MP . Frequency and outcomes of primary central sleep apnea in a population‐based study. Sleep Med. 2020;68:177‐183. doi:10.1016/j.sleep.2019.12.008 32044555 PMC9272740

[38] Liguori C , Cremascoli R , Maestri M , et al. Obstructive sleep apnea syndrome and Alzheimer's disease pathology: may continuous positive airway pressure treatment delay cognitive deterioration?. Sleep Breath. 2021;25(4):2135‐2139. doi:10.1007/s11325-021-02320-4 33619666

[39] Skiba V , Novikova M , Suneja A , McLellan B , Schultz L . Use of positive airway pressure in mild cognitive impairment to delay progression to dementia. J Clin Sleep Med. 2020;16(6):863‐870. doi:10.5664/jcsm.8346 32039755 PMC7849660

[40] Wang Y , Cheng C , Moelter S , et al. One year of continuous positive airway pressure adherence improves cognition in older adults with mild apnea and mild cognitive impairment. Nurs Res. 2020;69(2):157‐164. doi:10.1097/nnr.0000000000000420 32108738 PMC7212768

[41] Berlowitz DJ , Shafazand S . CPAP and cognition in OSA (APPLES). J Clin Sleep Med. 2013;9(5):515‐516. doi:10.5664/jcsm.2682 23674945 PMC3629328

[42] Kushida CA , Nichols DA , Holmes TH , et al. Effects of continuous positive airway pressure on neurocognitive function in obstructive sleep apnea patients: the apnea positive pressure long‐term efficacy study (APPLES). Sleep. 2012;35(12):1593‐1602. doi:10.5665/sleep.2226 23204602 PMC3490352

[43] Bliwise DL , Greenaway MC . Will APPLES hit a ceiling?. Sleep. 2011;34(3):249‐250. doi:10.1093/sleep/34.3.249 21358840 PMC3041699

[44] Rast P , Hofer SM . Longitudinal design considerations to optimize power to detect variances and covariances among rates of change: simulation results based on actual longitudinal studies. Psychol Methods. 2014;19(1):133. 24219544 10.1037/a0034524 PMC4080819

[45] Kasper JD , Freedman VA , Spillman B . Classification of persons by dementia status in the National Health and Aging Trends Study. Technical Paper #5. Baltimore: Johns Hopkins University School of Public Health. 2013. Available at https://www.nhats.org

[46] Chen D , Sekhon VK , Roth DL , et al. Harmonizing late‐life cognitive performance data across two population‐based cohort studies: the health retirement study and National Health and Aging trends study. Alzheimers Dement. 2025;17(4):e70198. 10.1002/dad2.70198 PMC12516223 41089236

[47] Freedman VA , Schrack JA , Skehan ME . National Health and Aging Trends Study User Guide: Rounds 1‐14 Final Release. Baltimore: Johns Hopkins School of Public Health. 2026. Available at https://www.nhats.org

[48] National Center for Health S, Bureau USC . Household pulse survey, 2020‐2024: anxiety and depression. 2025/09/22 2025.

[49] Laird NM , Ware JH . Random‐effects models for longitudinal data. Biometrics. 1982;38(4):963‐974. 7168798

[50] Wickwire EM , Jobe SL , Oldstone LM , Scharf SM , Johnson AM , Albrecht JS . Lower socioeconomic status and co‐morbid conditions are associated with reduced continuous positive airway pressure adherence among older adult medicare beneficiaries with obstructive sleep apnea. Sleep. 2020;43(12):zsaa122. doi:10.1093/sleep/zsaa122 32575113

[51] Wickwire EM , Fernandez CR , Huynh N , Watson NF , Duncan I . Association between positive airway pressure therapy and healthcare costs among older adults with comorbid obstructive sleep apnea and common chronic conditions: an actuarial analysis. Sleep. 2025;48(9):zsaf009. doi:10.1093/sleep/zsaf009 39803895

[52] Beydoun HA , Beydoun MA , Weiss J , et al. Insomnia as a predictor of diagnosed memory problems: 2006‐2016 health and retirement study. Sleep Med. 2021;80:158‐166. doi:10.1016/j.sleep.2021.01.038 33601227 PMC11000697

[53] Gottesman RF , Lutsey PL , Benveniste H , et al. Impact of sleep disorders and disturbed sleep on brain health: a scientific statement from the American heart association. Stroke. 2024;55(3):e61‐e76. doi:10.1161/STR.0000000000000453 38235581

[54] Lavie L . Oxidative stress—a unifying paradigm in obstructive sleep apnea and comorbidities. Prog Cardiovasc Dis. 2009;51(4):303‐312. doi:10.1016/j.pcad.2008.08.003 19110132

[55] Nair D , Ramesh V , Gozal D . Cognitive deficits are attenuated in neuroglobin overexpressing mice exposed to a model of obstructive sleep apnea. Front Neurol. 2018;9:426. doi:10.3389/fneur.2018.00426 29922222 PMC5996123

[56] Sullivan CE , Issa FG , Berthon‐Jones M , Eves L . Reversal of obstructive sleep apnoea by continuous positive airway pressure applied through the nares. Lancet. 1981;1(8225):862‐865. 6112294 10.1016/s0140-6736(81)92140-1

[57] Holth JK , Fritschi SK , Wang C , et al. The sleep‐wake cycle regulates brain interstitial fluid tau in mice and CSF tau in humans. Science. 2019;363(6429):880‐884. doi:10.1126/science.aav2546 30679382 PMC6410369

[58] Nedergaard M . Neuroscience. Garbage truck of the brain. Science. 2013;340(6140):1529‐1530. doi:10.1126/science.1240514 23812703 PMC3749839

[59] Djonlagic I , Saboisky J , Carusona A , Stickgold R , Malhotra A . Increased sleep fragmentation leads to impaired off‐line consolidation of motor memories in humans. PLoS One. 2012;7(3):e34106. doi:10.1371/journal.pone.0034106 22470524 PMC3314699

[60] Djonlagic IE , Guo M , Igue M , Kishore D , Stickgold R , Malhotra A . Continuous positive airway pressure restores declarative memory deficit in obstructive sleep apnea. Am J Respir Crit Care Med. 2021;203(9):1188‐1190. doi:10.1164/rccm.202011-4253LE 33347378 PMC8314910

[61] 2021 Alzheimer's disease facts and figures. Alzheimers Dement. 2021;17(3):327‐406. doi:10.1002/alz.12328 33756057

[62] DeVettori G , Troxel WM , Duff K , Baron KG . Positive airway pressure adherence among patients with obstructive sleep apnea and cognitive impairment: a narrative review. Sleep Med. 2023;111:28‐35. doi:10.1016/j.sleep.2023.08.029 37716335 PMC10613340

[63] Lajoie AC , Gu Y , Lim A , Benedetti A , Kaminska M . Adherence to continuous positive airway pressure for the treatment of obstructive sleep apnea in neurodegenerative diseases: a systematic review. Sleep Med Rev. 2023;71:101836. doi:10.1016/j.smrv.2023.101836 37586145

[64] Center for Medicare and Medicaid Services . CPAP for obstructive sleep apnea. Accessed October 16, 2021. https://www.cms.gov/Medicare/Coverage/Coverage‐with‐Evidence‐Development/CPAP

[65] Alpert N , Cole KV , Dexter RB , Sterling KL , Wickwire EM . Performance of claims‐based algorithms for adherence to positive airway pressure therapy in commercially insured patients with OSA. Chest. 2024;165(5):1228‐1238. doi:10.1016/j.chest.2024.01.020 38215934 PMC11214903

[66] Patel SR . Entering a new era in sleep‐apnea treatment. N Engl J Med. 2024;391(13):1248‐1249. doi:10.1056/NEJMe2407117 38912659

[67] Malhotra A , Grunstein RR , Fietze I , et al. Tirzepatide for the treatment of obstructive sleep apnea and obesity. N Engl J Med. 2024;391(13):1193‐1205. 38912654 10.1056/NEJMoa2404881 PMC11598664

[68] Food US, Drug A . FDA approves first medication for obstructive sleep apnea. 2024/12/20 2024.

