# Supplementary material for: Untreated obstructive sleep apnea and accelerated cognitive decline over 10 years
Source: Alzheimers Dement. 2026 Jul 14;22(7):e71533. doi: 10.1002/alz.71533 (PMC13366499; doi:10.1002/alz.71533)
Supplement: Supplementary file 1 — Supporting Information [file ALZ-22-e71533-s001.docx]

**SUPPLEMENTARY MATERIALS**

**Table S1.** Variance estimates for Model 2 analyses

|  | **Variance** | | |  | **Relative Increase in Variance** | **Fraction Missing Information** | **Relative Efficiency** |
| --- | --- | --- | --- | --- | --- | --- | --- |
| **Parameter** | **Between** | **Within** | **Total** | **DF** |  |  |  |
| Intercept | 0.00035 | 0.056092 | 0.056477 | 194006 | 0.006858 | 0.006821 | 0.999318 |
| CPAP: Untreated vs Treated (ref) | 2.23E-06 | 0.001434 | 0.001436 | 3.07E+06 | 0.001714 | 0.001712 | 0.999829 |
| Time (per year) | 2.33E-08 | 3.84E-05 | 0.000038447 | 2.03E+07 | 0.000666 | 0.000666 | 0.999933 |
| Interaction: CPAP × Time | 3.47E-08 | 7.78E-05 | 0.000077868 | 3.74E+07 | 0.000491 | 0.000491 | 0.999951 |

**Table S2.** Variance estimates for Model 3 analyses

|  | **Variance** | | |  | **Relative Increase in Variance** | **Fraction Missing Information** | **Relative Efficiency** |
| --- | --- | --- | --- | --- | --- | --- | --- |
| **Parameter** | **Between** | **Within** | **Total** | **DF** |  |  |  |
| Intercept | 0.000408 | 0.062486 | 0.062935 | 177114 | 0.00718 | 0.00714 | 0.999287 |
| CPAP: Untreated vs Treated (ref) | 1.3E-06 | 0.001355 | 0.001356 | 8.05E+06 | 0.001058 | 0.001058 | 0.999894 |
| Time (per year) | 1.34E-08 | 3.73E-05 | 0.00003731 | 5.79E+07 | 0.000394 | 0.000394 | 0.999961 |
| Interaction: CPAP × Time | 1.54E-08 | 7.56E-05 | 0.000075616 | 1.80E+08 | 0.000224 | 0.000223 | 0.999978 |
